# An Active Role of the ΔN Isoform of p63 in Regulating Basal Keratin Genes K5 and K14 and Directing Epidermal Cell Fate

**DOI:** 10.1371/journal.pone.0005623

**Published:** 2009-05-20

**Authors:** Rose-Anne Romano, Kori Ortt, Barbara Birkaya, Kirsten Smalley, Satrajit Sinha

**Affiliations:** Department of Biochemistry, State University of New York at Buffalo, Center for Excellence in Bioinformatics and Life Sciences, Buffalo, New York, United States of America; Katholieke Universiteit Leuven, Belgium

## Abstract

**Background:**

One major defining characteristic of the basal keratinocytes of the stratified epithelium is the expression of the keratin genes K5 and K14. The temporal and spatial expression of these two genes is usually tightly and coordinately regulated at the transcriptional level. This ensures the obligate pairing of K5 and K14 proteins to generate an intermediate filament (IF) network that is essential for the structure and function of the proliferative keratinocytes. Our previous studies have shown that the basal-keratinocyte restricted transcription factor p63 is a direct regulator of K14 gene.

**Methodology/Principal Findings:**

Here we provide evidence that p63, specifically the ΔN isoform also regulates the expression of the K5 gene by binding to a conserved enhancer within the 5′ upstream region. By using specific antibodies against ΔNp63, we show a concordance in the expression between basal keratins and ΔNp63 proteins but not the TAp63 isoforms during early embryonic skin development. We demonstrate, that contrary to a previous report, transgenic mice expressing ΔNp63 in lung epithelium exhibit squamous metaplasia with de novo induction of K5 and K14 as well as transdifferentiation to the epidermal cell lineage. Interestingly, the in vivo epidermal inductive properties of ΔNp63 do not require the C-terminal SAM domain. Finally, we show that ΔNp63 alone can restore the expression of the basal keratins and reinitiate the failed epidermal differentiation program in the skin of p63 null animals.

**Significance:**

ΔNp63 is a critical mediator of keratinocyte stratification program and directly regulates the basal keratin genes.

## Introduction

Keratin proteins belong to two distinct classes, type I acidic and type II basic and represent the bulk of IF genes expressed in epithelial cells [1]. Type I and type II keratins are often co-expressed in specific pairs in both a tissue-specific and differentiation-specific fashion. For example, the pair formed by type I K14 and the type II K5 is characteristic of the mitotically active basal cells of the epithelium that line the stratified surface of the skin, digestive tract, genito-urinary tract and mammary glands among other tissues and organs. As the proliferating basal epithelial cells stop dividing and initiate a specialized program of differentiation, they down-regulate the expression of K5 and K14 and induce the expression of new sets of keratin genes depending upon the particular tissue in which they are located [2]. The restricted expression pattern of the basal-specific keratin genes is established during embryonic development and is thought to be controlled primarily at the transcriptional level. Indeed, during early embryonic morphogenesis, a switch from expression of simple epithelial keratins K8 and K18 to the stratified epithelial markers K5 and K14 is a critical developmental event that marks the stage of commitment and stratification of the epidermis [3], [4].

Our understanding of the molecular mechanisms that govern the transcriptional control of the basal keratin pair, K5 and K14, has increased over the last few years. By using DNAse I hypersensitive site mapping and reporter gene assays both in keratinocytes in culture and in transgenic animals in vivo, we and others have identified several cis-regulatory elements of the *K5* and *K14* genes, which are important for their cell-type specific expression [5], [6], [7], [8], [9], [10]. Many of these regulatory elements are scattered in the 5′ flanking regions of the *K5* and *K14* gene and are highly evolutionarily conserved. Biochemical studies of these DNA sequences have uncovered the role of several transcription factors such as AP-1, AP-2, Sp1 and Ets proteins in regulating K5 and K14 gene expression. Interestingly, many of these transcription factors are broadly expressed and not restricted to basal keratinocytes. This observation had led to the suggestion that combinatorial effects of ubiquitous transcription factors or restricted co-activators might regulate the basal-keratinocyte specific expression of K5 and K14. However, the identification of transcription factor p63, which is highly restricted to basal keratinocytes and directly regulates *K14* gene expression by interacting with an enhancer element has rekindled renewed interest in this field [11].

The transcription factor p63, a homolog of the tumor suppressor p53, plays an important role in maintaining the progenitor basal cell populations in stratified epithelium such as those in the epidermis of the skin [12]. Indeed, p63-null mice exhibit a striking block in skin development [13], [14], [15]. In the absence of p63, the embryo is encased in a primordial epithelial layer that fails to express the basal markers K5 and K14 and does not undergo proper stratification and differentiation. The skin phenotype observed in p63^−/−^ mice has led to the formulation of two hypotheses concerning the biological role of p63 in epidermal development [16], [17]. According to one hypothesis, p63 may be important in maintaining the proliferative potential of the stem cell population in the epithelium [18]. Conversely, studies from a different independently generated p63 null mouse model suggest that p63 may be required in the commitment step to the stratified epithelial lineages [19]. Notwithstanding these differences, it is clear that p63 is a critical transcriptional regulator required for keratinocyte development and differentiation [20]. One quirk that has compounded a thorough and careful analysis of this transcription factor is the myriad naturally occurring isoforms of p63.

The *Trp63* gene encoding p63 gives rise to multiple functionally distinct protein isoforms, including TAp63, which encodes for an amino terminal transactivation domain (TA) and ΔNp63, which lacks the N-terminal transactivation domain (ΔN), and is synthesized from an internal promoter [21]. In addition, both TA and ΔN transcripts are differentially spliced at the 3′ end generating proteins with unique C-termini, which are designated as α, β, and γ isoforms. Unlike the shorter β and γ isoforms, the p63α isoforms harbour a Sterile Alpha Motif (SAM) domain, which is thought to mediate protein-protein interactions. One controversy regarding p63 has been fueled by the conflicting observations regarding the expression and function of the two major N-terminal variant isoforms, primarily during epidermal development. While it is unambiguous that ΔNp63 is highly expressed in basal keratinocytes, the expression profile of TAp63 remains unclear. Earlier studies have suggested that TAp63 and not ΔNp63 is the primary driver of the switch in expression of simple epithelial keratins K8 and K18 to the stratified epithelial markers K5 and K14 during early embryonic morphogenesis [19]. However, the fact that undisputed identification of TAp63 mRNA and protein in mouse embryonic skin remains elusive and that the TAp63 isoform-specific knockout mice have no discernible skin phenotype warrants a re-evaluation of this observation [22]. Since the ΔNp63 isoforms lack the amino-terminal sequences, it was originally proposed that these proteins might act primarily as dominant negative regulators [21]. However recent studies indicate that ΔNp63 proteins have intrinsic biological activities and can transcriptionally activate or repress target gene expression [23], [24], [25], [26], [27], [28]. Taken together, these findings have now shifted the attention towards ΔNp63 and its perceived role in actively dictating early epidermal development and governing keratinocyte cell fate and lineage choices.

To address the expression profile of the two major isoforms of p63, in the present study, we have utilized ΔN and TA specific antibodies. Our studies confirm that in the early stages of skin development, ΔNp63 proteins predominate and are hence likely to mediate the commitment of ectodermal cells to an epidermal cell fate. In support of this hypothesis we have identified a p63-response element present in a highly conserved enhancer corresponding to a DNAse I hypersensitive segment of the basal keratinocyte restricted *K5* gene. We provide biochemical evidence that ΔNp63 can interact with and activate the K5 enhancer and that all ΔNp63 isoforms are capable of inducing *de novo* expression of K5 in a cell line that does not express detectable levels of p63 or K5. Finally, we show that ectopic expression of various isoforms of ΔNp63 in transgenic mouse lungs and in p63 null animals, leads to *de novo* induction and restoration respectively, of both K5 and K14 gene expression. Taken together, our data provide additional insights into the regulatory mechanisms governing K5 gene expression, and suggest that K5, like its partner K14, is regulated in a direct and similar fashion by ΔNp63.

## Results

### Development of p63 isoform specific antibodies

**Figure 1:**
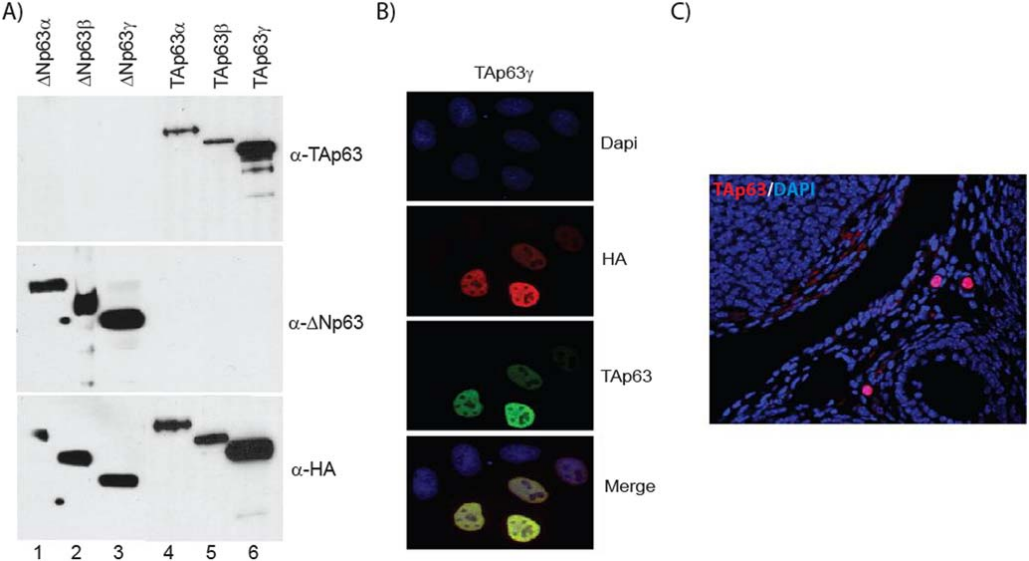
Generation of TAp63-specific antibody. A) Specificity of the TAp63 antibody. Western blot analysis of whole cell extracts transfected with various HA-epitope tagged isoforms of p63 show reactivity of the TAp63 antibody with cells transfected with HA-TAp63α, TAp63β, and TAp63γ only. B) Immunofluorescence of cells transfected with HA-TAp63γ demonstrate the ability of the TAp63 antibody to detect TAp63 in cell culture *in vivo*. DAPI nuclear staining is shown in blue, HA epitope tag is shown in red, TAp63 specific staining is shown in green and a merge is shown in the lower panel. C) TAp63 expression in oocytes in sections of mouse ovary. TAp63 expression is shown in red and DAPI is shown in blue.

Prior to this study, the expression analysis of ΔN and TA p63 isoforms during various stages of epidermal development has been performed using RT-PCR and in situ hybridization [19], [29]. However, a systematic and careful analysis of the respective proteins has not been undertaken with isoform-specific antibodies. To address this shortcoming, we generated TA-specific antibodies against the N-terminal portion of mouse TAp63 (amino acids 1–115), a domain that is not shared with ΔNp63. We performed Western blot analysis and immunofluorescence studies to address specificity of the anti-TA antibodies. As shown in Figure 1A, the anti-TAp63 antibodies detected the proteins at the expected sizes only in HA epitope-tagged TAp63α, β, or γ transfected cells but did not detect any band in the HA epitope-tagged ΔNp63 α, β, or γ transfected cells, confirming the specificity of the newly generated polyclonal antibodies. Western blot with anti-ΔNp63 (RR-14) antibodies demonstrated that the ΔNp63 proteins were indeed expressed in the transfected cells and did not cross-react with TA proteins as previously demonstrated. The expression of all six isoforms of p63 proteins was verified by western blot with anti-HA antibodies. Immunofluorescence studies further validated these data, since anti-TA antibodies recognized TAp63 protein localized predominantly in nuclei of transiently transfected HeLa cells (Figure 1B). Finally, to examine the affinity of the anti-TAp63 polyclonal antibodies to TAp63 proteins in vivo, we performed immunoflourescence on ovary sections where TAp63 has been shown to be highly expressed. The staining pattern observed in the ovarian follicles using our anti-TA antibodies matched exactly with published reports, further confirming the specificity of our newly developed antibodies (Figure 1C) [22].

### Expression analysis of p63 proteins during embryonic skin development reveals a predominance of the ΔNp63 isoforms

**Figure 2:**
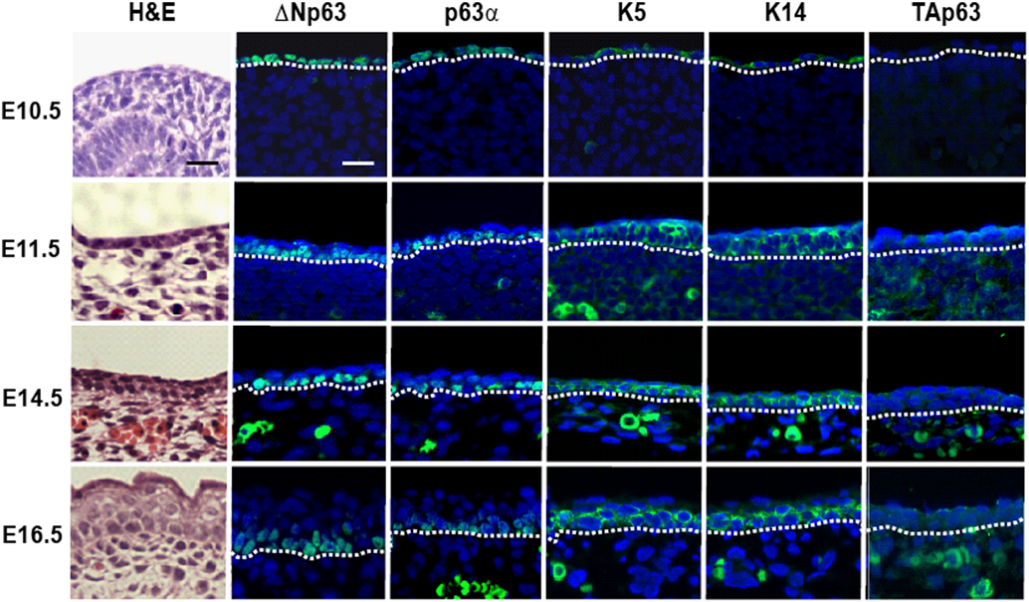
ΔNp63 is expressed during early mouse embryonic skin development. Isoform specific expression of p63 was analyzed during various stages of skin development using three antibodies recognizing different p63 variants. Far left panel (panel 1) are H&E staining of skin using paraffin embedded whole embryos. Panel 2 demonstrates expression of ΔNp63 (green) using the RR-14 antibody. Panel 3 shows expression of p63α (green) using the H-129 antibody. K5 and K14 expression is also shown in green (panels 3 and 4 respectively). Far right panel reveals that TAp63 expression is absent during the developmental windows examined. Scale bar: 50 µm.

Having confirmed the specificity of the antibodies against the two isoforms of p63, next we examined mouse embryonic sections at different stages of development. At early embryonic day 10.5 and 11.5 (E10.5 and E11.5), the primitive epithelial lining encasing the embryo expressed ΔNp63 as shown by a strong and uniform nuclear staining with anti-ΔNp63 RR-14 antibodies (Figure 2). Similar staining was observed with H-129 antibodies, which recognize an epitope in the C-terminus of the longer p63α isoforms. We also performed immunofluorescence with anti-K5 and anti-K14 antibodies and observed similar overlapping expression profile to that of ΔNp63 except the staining was cytoplasmic as expected. However, antibodies against TAp63 failed to show any staining suggesting that under similar experimental conditions, the TAp63 proteins are either not expressed or expressed at levels undetectable by our antibodies. To follow the developmental pattern of the expression of p63 isoforms and the basal keratins, we performed additional immunofluorescence studies at E14.5 and E16.5, two stages where the epidermal stratification and differentiation programs are distinctly matured as evident by histology (Figure 2, left panel). Similar to results obtained at E11.5, at these later stages both RR-14 and H-129 antibodies detected strong ΔNp63 expression in the basal cells of the stratified epidermis, whereas TAp63 was undetectable. These results thus indicated that TAp63 proteins are virtually non-existent in the developing skin epidermis, whereas ΔNp63 and the K5/K14 keratin pair show strong and overlapping expression in the basal keratinocytes at different stages of embryonic development.

### A hypersensitive site located upstream of the K5 gene is evolutionarily conserved and is a direct transcriptional target of p63

Recently we have shown that K14 expression is induced and directly regulated by ΔNp63 [11]. Based on the fact that K5 is co-expressed with K14 and likely to be coordinately regulated, we hypothesized that K5 is also a direct transcriptional target of p63. To test this hypothesis, we searched the genomic sequences in and around the *K5* gene for potential p63 binding sites. Previously, using DNase I Hypersensitive (Hs) site mapping, we had identified several Hs located within the 5′ region of the *K5* gene and shown that these elements act as enhancers important for the transcriptional control of *K5* gene expression [6]. Hence we targeted our search for p63-response elements (p63RE) in these potential enhancer elements based on sequence similarities to the p63 consensus site as defined by a number of laboratories, including our own [30], [31], [32], [33]. Although several potential p63REs were identified, one specific element embedded in Hs II region and located ∼ −960 bp from the transcriptional start site showed the highest level of sequence similarity. In addition, sequence alignment of Hs II, demonstrated that this region was evolutionarily conserved between human, mouse, cow, dog and oppossum, suggesting the possibility of a functionally important role for this DNA element (Figure S1). Next, we asked if the p63RE was capable of interacting with ΔNp63.

### The transcription factor ΔNp63 binds to p63RE DNA sequences located within K5 Hs II region

**Figure 3:**
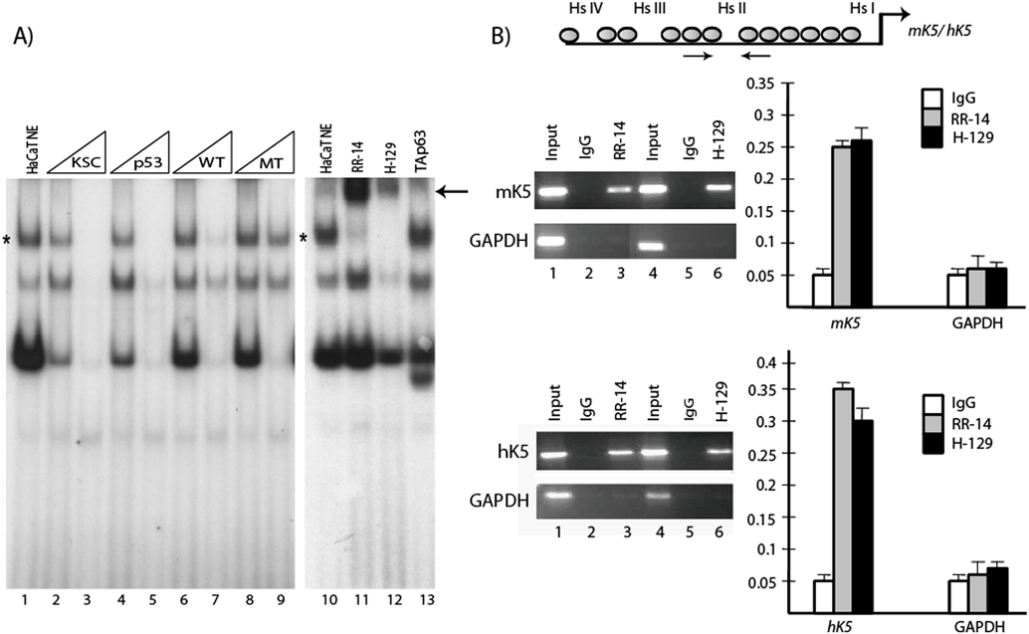
Binding of ΔNp63α to Hs II region of the *K5* gene. A) EMSA reveals binding of ΔNp63α in nuclear extracts of HaCaT cells using a radiolabeled probe corresponding to Hs II of the human K5 gene. Competition assays show that the KSC oligonucleotide, which contains a previously identified ΔNp63α binding site within the K14 gene, competes with binding of Hs II probe (lanes 2–3). A p53 consensus (lanes 4–5) and a WT oligonucleotide (lane 6–7) also compete for binding while a MT oligonucleotide did not affect Hs II binding (lanes 8–9). The highest complex (asterix) is supershifted (arrow) with the addition of antibodies raised against various domains of ΔNp63 whereas anti-TA antibodies have no effect (lanes 10–13). The lower complexes are likely to be non-specific. B) *In vivo* occupancy of ΔNp63α to the mouse and human *K5* gene. ChIP was performed on HaCaT or primary mouse keratinocytes using two separate antibodies recognizing ΔNp63α as well as a nonspecific IgG as indicated (left panel). Input represents PCR amplification of 1% of the genomic DNA. Primers corresponding to a region of the GAPDH gene serves as a negative control. Right panel shows results obtained from real-time PCR experiments.

In order to test if the K5 Hs II segment contains a *bona fide* p63 binding site, we performed Electrophoretic Mobility Shift Assays (EMSA). Using a radiolabeled oligonucleotide probe corresponding to this putative p63 binding site, we showed specific DNA-protein complexes with nuclear extracts from HaCaT cells, an immortalized human keratinocyte cell line which has been shown to express high levels of ΔNp63 (Figure 3, lane 1). In order to determine if ΔNp63 comprised this protein complex, we performed competition assays. We utilized a number of different probes including a previously identified p63 binding sequence located within the K14 enhancer (KSC), a consensus p53 sequence known to bind p63 (p53), a wild-type (WT), and a mutant (MT) double-stranded oligonucleotide. While increasing amounts of the KSC, p53, and WT oligonucleotides disrupted binding of the complex, the MT oligonucleotide was unable to compete (Figure 2 lanes 2–9). These results suggested to us that the protein-DNA complex likely consisted of p63.

To further confirm the identity of this nuclear complex, supershift experiments were performed. The addition of an antibody that specifically recognizes the ΔNp63 isoform of p63 (RR-14) results in a clear supershift (Figure 3, compare lanes 10 to 11). We also used a second commercially available antibody (H-129), and observed a similar supershift of the complex (Figure 3, lane 12). On the contrary, anti-TA antibodies did not effect the p63-DNA complex in agreement with the fact that these keratinocytes do not express any detectable TAp63.These results strongly suggest that the ΔNp63 and not the TA isoform of p63 binds to the specific p63RE present in Hs II of the K5 gene.

### Occupancy of K5 Hs II by ΔNp63α in keratinocytes

In light of the results obtained from our *in vitro* experiments, we next wanted to verify whether ΔNp63 was able to interact with Hs II of the *K5* gene *in vivo*, by performing ChIP assays in human keratinocytes. Two separate antibodies against p63, one directed specifically against ΔNp63 (RR-14) and one that recognizes the p63α isoforms (H-129), as well as a control antibody (IgG) were used for the ChIP experiments. To determine if ΔNp63 is associated with this region, we used primers, which specifically amplify Hs II region. As a negative control, primers that amplify a genomic segment corresponding to gyceraldehyde-3-phosphate dehydrogenase (GAPDH) were chosen. Our PCR data clearly shows a specific amplification of the Hs II region in p63-immunoprecipitated genomic DNA (Figure 3B). We further quantified this result by real time PCR, which demonstrated a 6.6, and 5-fold enrichment of Hs II in samples immunoprecipitated with RR-14 and H-129 respectively, versus control antibody (Figure 3B). In contrast we did not observe any recruitment of ΔNp63 to the control region corresponding to the GAPDH gene. Due to the high level of sequence conservation observed for Hs II between both human and mouse, we wondered whether ΔNp63 similarly occupied this region in the mouse *K5* gene. We therefore repeated our ChIP experiments utilizing primary mouse keratinocytes. There was a specific localization of p63 to the mouse K5 5′ genomic segment as evident by both regular PCR as well as real time PCR results (Figure 3B). Taken together, our data demonstrate that the ΔNp63α isoform of p63 binds to Hs II of the *K5* gene in both human and mouse keratinocytes *in vivo,* supporting the possibility of a functional role for this p63 isoform in the regulation of *K5* gene expression.

### Identification of an enhancer element corresponding to Hs II within the *K5* gene

Having demonstrated the occupancy of ΔNp63 to Hs II of the K5 gene, we next investigated whether this segment could function as an enhancer element. To determine the contribution of Hs II element to keratinocyte-specific expression of K5, we assayed for the ability of this element to activate transcription of a luciferase (*luc*) reporter gene in transient transfection assays. We therefore generated a construct in which the sequences corresponding to Hs II were cloned upstream of the K5 minimal promoter and luciferase reporter gene (HsIIK5Luc). In our transfection assays using primary mouse keratinocytes, we found that HsIIK5Luc is able to activate transcription 7-fold over the K5 minimal promoter alone (K5Luc) (Figure S2A). A CMVLacZ plasmid was co-transfected to serve as an internal control for transfection efficiency. To test if the transcriptional activation of Hs II was also observed with a heterologous promoter, we generated an additional construct where Hs II was cloned upstream of the minimal promoter of thymidine kinase (TK) (HsIITKLuc). As shown in Figure S2, HSIITKLuc showed a 3-fold activation over the TK minimal promoter alone (TKLuc). These data confirm the ability of HS II to act as an enhancer element, likely contributing to the keratinocyte-specific expression of K5.

Finally to test the ability of various p63 isoforms to activate the K5 enhancer element, we performed transient transfection experiments in Ptk2 cells, which do not express endogenous p63 or K5. The HsIIK5Luc construct was transfected into Ptk2 cells along with either a control expression plasmid pCMVHA, or plasmids expressing various HA-epitope tagged isoforms of p63. Again, a CMVLacZ plasmid was co-transfected to serve as an internal control for transfection efficiency. As data presented in Figure S2B indicate, expression of ΔNp63α, ΔNp63β, and ΔNp63γ isoforms led to 3–7 fold higher transcriptional activity of the K5 enhancer element respectively, as compared to the empty vector alone. Under similar conditions, activation by TAp63α, TAp63β, and TAp63γ isoforms was modest and showed only 1.5–2 fold higher transcriptional activity of the K5 enhancer element, as compared to the empty vector alone. These data suggest that various ΔNp63 isoforms can regulate the expression of the K5 enhancer at the transcriptional level and this activation is considerably more potent than the TAp63 isoforms.

### Ectopic expression of multiple isoforms of p63 can induce expression of K5

**Figure 4:**
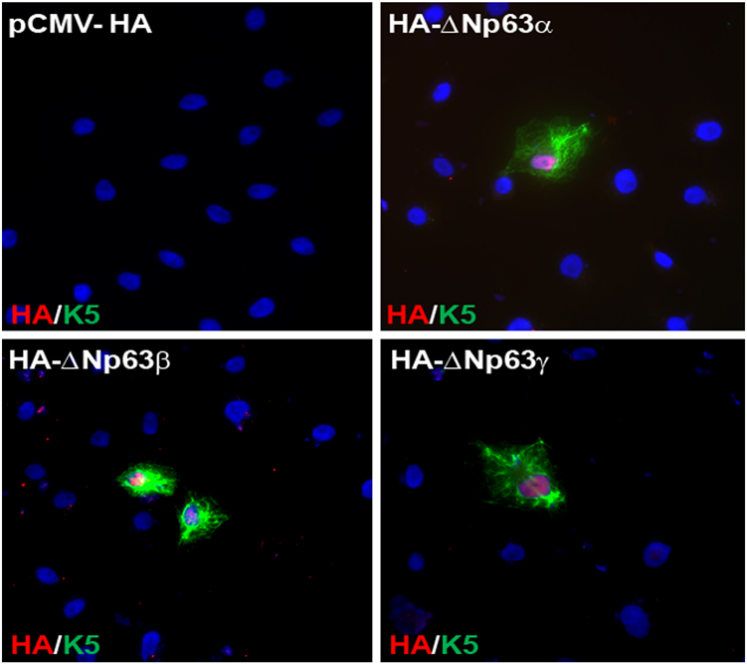
Three major isoforms of ΔNp63 can induce expression of K5 in cell culture. Ptk2 cells were transfected with plasmids encoding different HA-epitope tagged isoforms of ΔNp63 as indicated. Cells were stained with antibodies detecting HA (red) and K5 (green). Nuclei are stained with DAPI (blue). 40× magnification is shown.

We have previously shown that ectopic expression of the various isoforms of ΔNp63 are capable of inducing K14 expression and that this induction is dependent upon the DNA-binding activity of p63. In light of this finding, we wondered whether ΔNp63 was also capable of transactivating *K5* gene expression in cells grown in culture. To test this hypothesis, we transfected Ptk2 cells with expression plasmids encoding various HA-epitope tagged isoforms of ΔNp63 and evaluated induction of the endogenous *K5* gene. Immunofluorescence studies reveal that cells expressing all three ΔNp63α, ΔNp63β, and ΔNp63γ isoforms, as seen by anti-HA nuclear staining shown in red, also express the cytoplasmic network of K5 staining, shown in green (Figure 4). Nuclei were counterstained with 4′,6-diamidino-2-phenylindole (DAPI) shown in blue. Taken together, our studies demonstrate that ectopic expression of all three isoforms of ΔNp63 is capable of inducing expression of both K5 and K14 gene expression [11], [34].

### Expression of various isoforms of ΔNp63 in transgenic mice induces K5 and K14 expression

**Figure 5:**
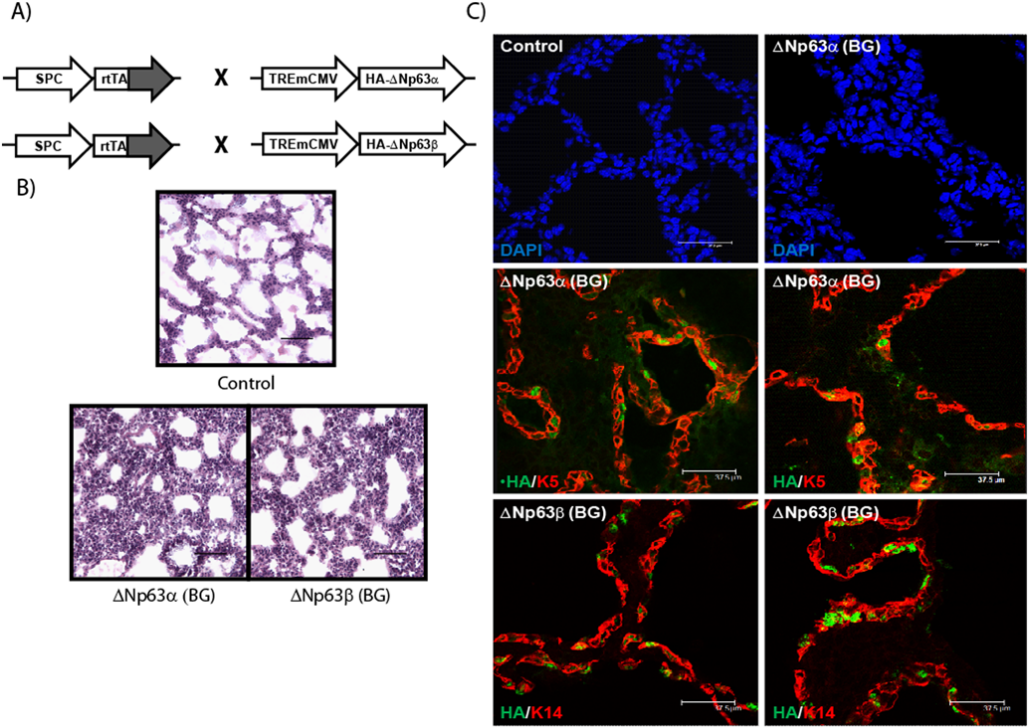
ΔNp63 can induce *de novo* expression of K5 and K14 in single-layered lung epithelia. A) Schematic depicting the mating scheme used to generate transgenic animals expressing the HA-ΔNp63 transgene in single-layered lung epithelium using the surfactant protein C (SPC) promoter in a tetracycline inducible fashion. B) Gross morphology of E18.5 lung tissue sections stained with H&E from control animals and bi-transgenic animals (ΔNp63αBG and ΔNp63βBG). Bi-transgenic animals exhibit squamous metaplasia. Scale bar: 50 µm. C) Lung tissue sections from E18.5 stained with antibodies detecting HA epitope tag (green) and K5 or K14 (red) reveal expression of the transgene in bi-transgenic animals. Dapi staining is shown in blue. Tissues from bi-transgenic animals demonstrate induction of K5 and K14 expression (red staining) as compared to control littermates.

To determine whether the ΔNp63 isoforms of p63 are capable of inducing *K5* and *K14* gene expression *in vivo*, we ectopically expressed HA-epitope tagged ΔNp63α or ΔNp63β, under the control of a tetracycline-inducible promoter (HA-ΔNp63α or HA-ΔNp63β) in simple lung epithelia. Transgene expression was directed to lung epithelia using mice expressing the reverse tetracycline-activator (rtTA) from the human Surfactant Protein-C (SPC) promoter (SP-C/rtTA). HA-ΔNp63α or HA-ΔNp63β transgenic mice, were bred to the SP-C/rtTA transgenic mice, which have been previously shown to regulate doxycycline-inducible expression of transgenes to single-layered lung epithelial cells [35]. The SP-C promoter targets transgene expression to the cuboidal surfactant-secreting type II alveolar cells, and to the distal Clara cells of the lung epithelium. Neither of these cell types expresses endogenous p63, K5, or K14. Doxycycline was administered at embryonic day 0.5 (E0.5) of gestation to induce transgene expression in the lung epithelia of the Bi-transgenic (BG) mice (ΔNp63αBG or ΔNp63βBG). Lung biopsy specimens obtained from E18.5 animals were stained with hematoxylin and eosin. The staining data demonstrated that ectopic expression of both the ΔNp63 isoforms resulted in severe alterations in lung alveolar epithelial morphology, including squamous metaplasia in BG animals as compared to WT control littermates (Figure 5). Moreover, immunofluorescence staining revealed that sustained expression of both ΔNp63α and ΔNp63β to the distal airway epithelium of the lungs, induced *de novo* expression of both K5 and K14. Staining of the lung tissue sections with HA antibody allowed us to confirm the ectopic expression of the ΔNp63 proteins in the lung epithelial cells. In addition, we also examined expression of Thyroid Transcription Factor I (TTF1), a known marker of the lung epithelial cells targeted by the SP-C promoter, which showed overlapping expression with ΔNp63 (data not shown) [36]. As shown in ure 5C, lung epithelial cells expressing the ΔNp63 transgene (green) also strongly expressed both K5 and K14 (red). This is in stark contrast to lung sections from wild type control littermate animals, which do not express the transgene and not show any staining for the basal keratins (data not shown). Interestingly, a subpopulation of K5 and K14+ve lung cells did not show detectable levels of p63, this could be possibly due to differences in protein stability or detection sensitivity or to unknown cell non-autonomous effects of the ectopically expressed ΔNp63. Taken together, these results indicate that ectopic expression of both ΔNp63α and ΔNp63β are sufficient to commit a single-layered epithelium to an epidermal cell fate. To examine whether these altered lung cell types expressing ΔNp63 further commit to a keratinocyte differentiation program, we stained BG lung tissues with antibodies against K1 and K10, which are characteristic markers of skin keratinocytes of the differentiated spinous layer. Surprisingly, BG lung epithelium, unlike WT control lungs exhibited strong expression of K1/K10 further validating the notion that expression of ΔNp63 alone was sufficient to trigger the transformation of lung cells along a stratifying epidermal pathway (Figure S3).

### Rescue of p63-null skin phenotype by ΔNp63 expressing transgenics using a Tet inducible system

**Figure 6:**
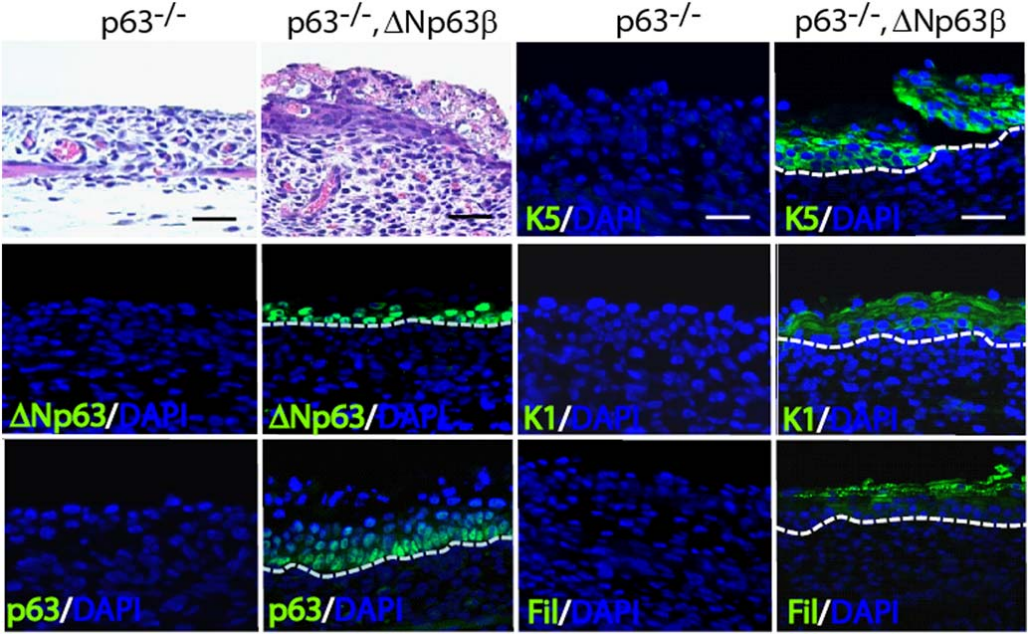
Ectopic Expression of ΔNp63β can partially rescue the p63 null phenotype. Top left panel shows H&E staining of p63^−/−^ and p63^−/−^,ΔNp63β rescued animals. Scale bar: 50 µm. Remaining panels illustrate immunofluorescence staining using various antibodies as indicated (in green). White hashed line demarcates the dermal epidermal boundary. Scale bar is 25 µm for immunofluorescence images.

TP63-deficient animals that lack all isoforms of p63 display a severe skin phenotype and die soon after birth. One previous study has examined the effects of restoring the expression of ΔNp63α or TAp63α or both these isoforms together in a p63 null animal by using the K5 promoter [37]. Interestingly, a partial rescue was observed in p63 null animals, which expressed ΔNp63α but not with TAp63α. Having established the Tet-inducible system, we re-examined the effects of over expression of ΔNp63 in the p63 null background. Our experimental plan is different from the published study on several accounts. First, we overexpressed ΔNp63 in the basal skin keratinocytes in an inducible fashion by utilizing the K5-promoter-Tet driver animals and second, the p63 knockout allele used in our study is different from the one previously used. This is important since it has been reported that the two p63 knockout alleles exhibit subtle but distinctly different skin phenotypes [13], [14]. Finally, we tested both ΔNp63α and ΔNp63β for their potential efficiency and functional role in the in vivo rescue experiments. Bi-transgenic animals that expressed either ΔNp63α or ΔNp63β did not completely restore the epidermal integrity of the p63 null animals as the animals perished soon after birth. However, upon histological examination, several areas were found to contain properly developed and stratified epidermis (Figures 6 and S4). Upon immunoflourescent staining, we observed strong expression of the basal markers K5 and K14, a feature completely absent from the p63 null newborn skin. Furthermore, staining these areas with the antibodies against p63 confirmed that p63 was indeed expressed in these focal areas and was confined to the basal layer. In agreement with the histological features, additional markers of differentiation such as K1, K10 (spinous layer) and filaggrin (granular layer) were also prominently expressed in focal areas of fully matured epidermis. Collectively, these data confirm results obtained from prior studies and suggest that the ΔNp63α and ΔNp63β isoforms alone are competent in initiating the expression of basal markers and stratification.

## Discussion

During embryonic development, the commitment of the epithelial cells to a stratified epidermal lineage is thought to initiate with the induction of the basal keratin pair K5 and K14 [38]. The association of the K5/K14 IF network with the proliferating basal keratinocytes of the skin is a distinguishing feature that persists throughout the life of an organism. One path to a better understanding of the regulatory mechanisms that govern the initial commitment of epithelial cells to a keratinocyte cell fate and subsequent stratification and differentiation involves identification of transcription factors that control the regulation of K5 and K14. Our prior studies identified a number of evolutionarily conserved DNase I Hs elements scattered in the 5′ region of the K5 and K14 gene. We had shown that various transcription factors including those belonging to AP-1, AP-2 and Ets family were important in mediating the activity of K5 and K14 enhancers [5], [6], [7]. However, recent studies have shown that in addition to these factors, the transcription factor p63 directly regulates K14 gene expression and plays a central role in the development of the keratinocyte lineage [11], [37].

Despite the growing evidence of the important role of p63 in skin biology, one of the main unresolved issues revolves around the expression profile, transcriptional activity and biological role of its two major isoforms, ΔNp63 and TAp63. Initial studies, similar to those described in this paper led to an interesting hypothesis, mainly that TAp63 isoforms are required for commitment to a stratified cell lineage during embryonic skin development [19], [39]. This “commitment” model further suggests that the dominant negative ΔNp63 function is required to oppose TAp63 isoforms to achieve proper differentiation. According to this study, TAp63 was expressed in the early ectodermal cells prior to the simple epithelial to epidermal switch. Furthermore it was capable of inducing the expression of K14 when ectopically expressed in epithelial cells and causing a squamous metaplasia when expressed in lung epithelium of transgenic mice. Indeed, the argument that had supported the notion that TAp63 was the driver for commitment was the fact that under similar experimental conditions, ΔNp63 failed to induce basal keratins both in cell culture and in transgenic mice.

Our data presented in this paper clearly argues against the model in which TAp63 proteins are the only isoforms that are competent in conferring a basal-cell like phenotype. We posit that any question about the functional relevance of p63 isoforms should begin with a clear understanding of where they are expressed. While the expression of TAp63 and ΔNp63 has been evaluated at the mRNA level by RT-PCR and by in situ hybridization experiments, the results have been contradictory. While according to one report, TAp63 mRNA can be detected in whole embryos as early as E9.5, other studies suggest that while in embryonic skin ΔNp63 mRNA is expressed at high levels, TAp63 mRNA could barely be detected [19], [29]. This discrepancy has been attributed to experimental differences and due to the difficulties in processing and manipulating mouse embryos at such early developmental stages. With this controversy plaguing the p63 field, it is hence very important that the relative expression of these two isoforms be clearly evaluated, in particular at the protein level.

We have now thoroughly evaluated the expression of TAp63 and ΔNp63 in mouse embryonic skin using antibodies generated in our laboratory that are specific to each isoform. These studies unequivocally argue that the ΔNp63 and not TAp63 isoforms are predominantly expressed at various stages of early embryonic skin development and co-localize with the basal keratin markers K5 and K14. One of the arguments that have been made is that the TA proteins are labile and hence notoriously difficult to detect [40], [41]. While this remains a possibility, our immunoflourescence studies with anti-TA antibodies clearly show robust expression of TAp63 in ovary. This data is also in agreement with the results obtained from the McKeon lab [22]. Interestingly, some recent publications have reported expression of TAp63 in the suprabasal layer of adult human skin [42], [43]. Using our anti-TAp63 antibodies, so far we have failed to detect TAp63 in the differentiated mouse keratinocytes — this could be due to species-specific or age related differences. Taken together, these results add to the growing body of evidence suggesting that under normal physiological conditions, ΔNp63 is the primary isoform expressed in keratinocytes.

Since p63 null animals lack epidermis and do not express K5 and K14, one potential role of p63 might be in regulating the expression of these basal-specific genes. Indeed, our studies have shown that p63 can directly interact with p63-responsive regulatory elements present in the enhancer of the K14 gene [11]. This data, coupled with the fact that p63 can activate or induce expression of K14 in a wide variety of epithelial cells strongly suggest that the basal keratin pair is a direct transcriptional target of p63 [11], [19], [34], [37], [44]. Despite the fact that K5 and K14 are typically co-expressed and hence likely to be coordinately regulated by a similar set of transcription factors, so far no experimental data have linked p63 to K5. To address this, we examined previously described DNAse I hypersensitive sites that are present in the 5′ upstream region of the K5 gene. Here we show that one of the most highly conserved enhancer segments does indeed contain a p63RE. We provide biochemical data to support the notion that ΔNp63 can bind to this element both by EMSA and ChIP experiments and that all three isoforms of ΔNp63 can induce K5 when ectopically expressed, thus confirming that K5, similar to its partner K14, is a direct transcriptional target of ΔNp63. Although we found no p63-response elements in the proximal promoter region of K5, we cannot rule out the recruitment of ΔNp63 to this region through protein-protein interactions with other transcription factors such as Sp1, as described for the ΔNp63 promoter for example [45].

Although our data clearly show that both the basal keratins K5 and K14 are direct p63 targets, there are some lingering questions. For example, it is disconcerting to note that the global ChIP studies on p63 targets have so far not identified the p63RE described in this study. This could be potentially due to an inherent limitation of the ChIP–Chip experiments in offering full coverage of the genome or differences in choice of cell lines, antibodies or other technical conditions. Hence, an argument can be made that the p63 target database is still evolving and far from completion, and there is a continuing need to find additional bona-fide p63 targets [46]. Another observation that warrants discussion is the result obtained from one knockdown study of p63, which showed no change in K5 and K14 levels in regenerated human tissue, in contrast to dramatic loss of these basal keratins in p63-null mice [41]. As suggested by the Khavari group, it is possible that regulation of the basal keratins differs in a developmental context versus that of committed, postnatal cells. Whereas the former process requires p63, the latter does not. Finally it is worth mentioning that expression of p63 in cell culture leads to a stronger and more consistent induction of K14 in contrast to K5 (our unpublished data). This is not surprising considering the growing evidence that the normal expression patterns of K5 and K14 in various basal cells might not be completely overlapping - a feature consistent with the notion that there might be differential mode of transcriptional regulation of K5 and K14 [47], [48], [49]. Interestingly, the induction of the basal keratins is not observed in a small percentage of p63+ve cells. This further suggests that the p63-mediated induction of specific genes is probably a complex process that is dependent upon a host of factors such as specific cell cycle stage, differentiation or epigenetic status, or the availability and activity of other transcription factors (such as AP-2) [50].

In an effort to probe the function of ΔNp63 in an in vivo setting, we have generated an inducible transgenic system to express two isoforms, ΔNp63α and ΔNp63β. We report that ectopic expression of ΔNp63 in the lung epithelium results in a dramatic switch in cell lineage commitment whereby the type II alveolvar cells show clear evidence of metaplastic changes from a simple epithelium to a stratified epithelium accompanied by robust induction of both K5 and K14 as well as K1 and K10. It has been shown that in the absence of p63, the stratified squamous epithelium in cervix and vagina is transformed into a simple columnar epithelium expressing uterine epithelial differentiation markers [15]. Similarly, the ventral epithelium of the p63^−/−^ bladder, where ΔNp63 is the predominant isoform is neither committed to stratification nor differentiated [51]. These observations taken with our gain-of-function studies further reinforce the idea that ΔNp63 is the primary instructive driver in maintaining a stratified and differentiated epithelium. The discrepancy between our findings and those from a prior study where ΔNp63 failed to activate basal-specific gene expression is likely due to differences in experimental set up or unknown modifying factors that remains to be sorted out.

One recent report has examined the relative contribution of ΔNp63 and TAp63 by reintroducing these isoforms separately or in combination in p63 null mice with the help of K5 promoter-driven transgenic animals that express ΔNp63 and TAp63 [37]. These studies by Candi et al. suggest that only ΔNp63 and not TAp63 when expressed under the K5 promoter can partially rescue the skin phenotype and that there is improved rescue when both ΔNp63 and TAp63 are co-expressed. This additive effect might be due to unique and complementary functions of ΔNp63 and TAp63 or simply the results of an overall higher level of p63 due to the presence of both isoforms. Our inducible transgenic model system allowed us to revisit this issue. One major difference in the two studies is that we used a different p63 null allele – an important consideration since it has been reported that these two animals show subtle yet distinct differences in the extent of epidermal integrity. While for the experiments described by Candi et al, p63 null animals supposedly contain clumps of differentiated cells in the epidermis, in the p63 null animals described in this study, no such clumps were observed in new born animals, as has been reported [13], [14], [37]. However, in agreement with prior studies, expression of ΔNp63 can partially restore the p63 null skin phenotype and give rise to isolated, small pockets of stratified epithelium. These epithelial structures morphologically resemble a fully differentiated skin and express the entire battery of differentiation markers from basal-specific K5 and K14, early differentiation K1 and K10 to terminal differentiation filaggrin. What is remarkable is the fact that even the shorter ΔNp63β isoform, lacking a significant portion of the C-terminal domain is capable of rescuing the skin phenotype. This observation suggests that the SAM domain, a unique feature of p63 and p73 but not p53, is dispensable for the epidermal cell fate and stratification inducing properties of p63 – a finding that is further substantiated by the lung squamous metaplasia phenotype of the SpC-ΔNp63β transgenic animals. There are several potential reasons to account for only a partial and patchy rescue of the p63 null skin by ΔNp63 overexpression - the K5/14 promoter is probably not active at an early enough developmental stage, and more importantly, promoter activity might be severely attenuated in the p63 null background given the fact that p63 itself is a critical regulator of these basal-specific promoters. It is important to highlight that exogenous expression of ΔNp63 in ES-derived ectodermal cells efficiently differentiates them into keratinocytes and that in zebrafish, knockdown of ΔNp63 leads to a block in epidermal proliferation and development [34], [52]. These data coupled with the fact that TA-specific knockout mice develop normal skin add further credence to the idea that ΔNp63 might be the critical p63 isoform required for epidermal morphogenesis [22]. Although the tide has clearly turned in favor of ΔNp63, the final confirmation awaits the generation of ΔNp63-specific knockout mice.

## Materials and Methods

### Ethics Statement

All mouse experiments were approved by the State University of New York at Buffalo IACUC committee and carried out in accordance with relevant national guidelines.

### Generation of anti-TAp63 antibodies

A partial cDNA of mouse TAp63 (corresponding to the N-terminal amino acids 1–115 unique to the TA isoforms) was cloned into pGEX-5X-1 vector. Recombinant GST-TAp631-115 protein was purified from *E.coli* using the protocol as described before [53]. Immunization of NZW rabbits was carried out with recombinant GST-p63TA1-115 protein (Alpha Diagnostic). Serum obtained from the third round of bleed of animal no.13 showed the highest antibody titer and was used for subsequent purification and enrichment by antigen dependent affinity chromatography. For this purpose, a His-tagged p63TA1-115 protein was generated by cloning the corresponding partial cDNA of mouse TAp63 in pCOLD vector. The purification of His-p63TA1-115 protein was performed according to standard protocols described previously [30]. Purified His-p63TA1-115 was used to generate an affinity column by crosslinking it to Cyanogen bromide-activated Sepharose 4B beads, and serum containing the anti-p63TA antibodies was purified on it. After extensive washing, antibodies were eluted with elution buffer containing either KSCN or Glycine. Fractions with the highest antibodies as judged by western blot analysis against TAp63 were utilized for subsequent experiments.

### Preparation of nuclear extracts, and Electrophoretic Mobility Shift Assay (EMSA)

Nuclear extracts from HaCaT cells were prepared by standard methods as described before [45]. EMSA's were performed with 5 µg of nuclear extracts and end-labeled double-stranded oligonucleotides as previously described [30]. Oligonucleotide sequences used were as follows: K5HsII 5′-GGCGGCCTTCAGCCTGTATCAACACAT-3′, K5HsII Mut 5′-GGCGGCCTTCAGCCTATATCAACACAT-3′. Protein-DNA complexes were resolved by gel electrophoresis on 5% non-denaturing polyacrylamide gel in 1Χ TBE buffer at room temperature. The gel was dried and visualized by autoradiography after electrophoresis. Anti-p63 antibodies RR-14 and H-129 used for supershift experiments have been described before [45].

### Transient Transfections and Reporter Assays

MK (mouse keratinocytes) and Ptk2 cells were plated in six-well plates the day prior to transfection. Transfections were performed using Fugene 6 reagent (Roche) according to manufacturer's instructions. One microgram each of ΔNp63 and TAp63 expression plasmids and luciferase reporter constructs were transfected per well along with 0.25 µg of *CMVLacZ* plasmid DNA to serve as an internal control for transfection efficiency. Cells were harvested 48 hours post-transfection and reporter assays were performed as described previously [45]. The K5 Hs II luciferase reporter constructs were generated using pGL3 plasmid backbone containing the K5 or TK promoter that has been described before [6], [7]. The Hs II region was amplified using primers, Forward 5′ CTACAGGGTACCGTCCCG CTGCTTGGAACAGGGTGG -3′ and Reverse 5′ CTACAGGCTAGCCCGGTTCCCCAG CCCCCCAGGTGTG-3′ and cloned using Kpn I and Nhe I restriction enzymes.

### Immunofluorescence

Ptk2 cells were plated at 75,000 cells/well in 12-well cell culture plates lined with microscope cover glasses. Cells were transfected at 30% confluency with 1 µg of expression plasmids containing HA-epitope tagged mouse ΔNp63α, ΔNp63β, and ΔNp63γ. Forty eight hours post-transfection, cells were fixed on cover glasses with 4% paraformaldehyde for 10 minutes and washed in PBS for 30 minutes. Cells were permeabilized with 0.1% TritonX-100 (diluted in PBS) for 3 minutes, rinsed in PBS, and blocked for 2 hours with 5% BSA (in PBS). Samples were then incubated for 2 hours at room temperature with rat anti-HA (Roche) and rabbit anti-Keratin 5 (gift from Julie Segre) at 1∶500 dilution in 5% BSA. Cells were then washed several times in PBS for 30 minutes. Samples were further incubated for 45 minutes in secondary antibodies, anti-rat IgG Alexa 568 (Molecular Probes Inc. Eugene, OR) (1∶750 dil.), anti-rabbit IgG FITC (BD Biosciences) (1∶500 dil.), and 4′,6-diamidino-2-phenylindole (DAPI) (Molecular Probes) at room temperature. Cells were washed several times in PBS for 30 minutes and then briefly rinsed in tap water. Microscope cover glasses were then mounted on microscope slides.

Lung tissues from E18.5 mouse embryos were embedded in OCT and then frozen immediately on dry ice. Tissues were sectioned to 5 µm and fixed in cold methanol for 5 minutes. Slides were then washed in PBS three times for 5 minutes each. Tissues where then circled with a PAP pen and blocked for 1 hour at room temperature in 5% BSA, 0.1% TritonX-100 in PBS.

Whole new born animals were fixed overnight in 10% NBF, dehydrated, paraffin embedded and sectioned to 4 µm thickness. Slides were de-paraffinized and rehydrate through a graded alcohol series. Antigen retrieval was performed by boiling slides in a microwave for 20 minutes in antigen retrieval solution (10 mM sodium citrate, 0.05% Tween-20, pH 6.0). Slides were allowed to cool for 20 minutes and then rinsed briefly in PBS, circled with a PAP pen and then blocked in 5% BSA, 0.1% TritonX-100 in PBS for 1 hour.

The primary antibodies used at the indicated dilutions were ΔNp63 (RR-14) (1∶50; Romano et al. 2006), p63α (1∶50; Santa Cruz, H-129), K5, K14, K1, Filaggrin (1∶200; gift from Dr Julie Segre), K5 (1∶200; Covance, CA), TAp63 (1∶50), HA (1∶200; Roche), TTF1 (1∶200; GeneTex, TX). Secondary antibodies used were anti-rat IgG Alexa 568 (1∶750; Molecular Probes), anti-mouse IgG Alexa 488 (1∶250; Molecular Probes), anti-rabbit IgG Alexa 568 (1∶750; Molecular Probes), and anti-mouse IgG FITC (1∶250; BD Biosciences). When staining with mouse monoclonal antibodies, we used the reagents and protocol from the MOM Basic kit (Vector Labs). Slides were mounted using Vectashield Mounting Medium with DAPI (Vector Labs) viewed with a Nikon FXA Fluorescence microscope. Images were captured using a Nikon digital camera and analyzed using ImageJ, Adobe Photoshop, and Adobe Illustrator software. Lung images were captured using Leica confocal microscopy and analyzed as above.

### Generation of HA-ΔNp63α and HA-ΔNp63β Transgenic animals

The HA-ΔNp63α and HA-ΔNp63β constructs were generated by cloning each of the full length mouse ΔNp63α and full length mouse ΔNp63β cDNAs containing a 5′ HA epitope tag into the pTRE Tight plasmid (BD Bioscience). Transgenic mouse lines were generated by separately microinjecting the purified DNA constructs into fertilized mouse oocytes derived from a mixed genetic background (C3Hf/HeRos X C57BL/10 Rospd). Seven HA-ΔNp63α transgenic founder lines and nine HA-ΔNp63β transgenic founder lines were identified by PCR analysis of tail DNA. The following primers were used to genotype the HA-ΔNp63 founders: forward (5′- GGAGAATTCGAGCTCGGTACCCG – 3′) and reverse (5′ – CGCTATTCTGTGCGTGGTCTG – 3′). The founders were then crossed to K5-tTA mice to determine which of the founders express the transgene [54]. Detailed analysis of the Tet-p63 transgenic animals will be described in a separate manuscript.

### Animals

Protocols for mouse experimentation were performed according to SUNY at Buffalo and RPCI IACUC protocols. The SPC-rtTA, K5-tTA, and p63^+/−^ mice have been previously described [13], [35], [54]. Mice were mated with each other and noon of the day the vaginal plug was observed was considered E0.5. The pregnant SPC-rtTA dams were administered 2 mg/ml of doxycycline supplemented with 5% sucrose in their drinking water until embryos were collected at the indicated time. For rescue experiments, K5-tTA/HA-ΔNp63α/p63^+/−^ mice were mated to K5-tTA/HA-ΔNp63α/p63^+/−^ mice, in the absence of doxycycline, to generate K5-tTA/HA-ΔNp63α/p63^−/−^ animals.

### Western Blots

Hela cells were plated at 150,000 cells/well in six-well cell culture plates 24 hours prior to transfection. Cells were transfected using Fugene 6 transfection reagent with 1 µg of each expression plasmid encoding the various HA-epitope tagged p63 isoforms. Cells were harvested 48 hours post-transfection, washed in PBS, and centrifuged at 2,000 rpm for 5 minutes. Whole cell extracts were prepared by resuspending the pellets in Laemmli Sample Buffer (Bio-Rad). Approximately equivalent amounts of each sample was denatured at 96°C for 10 minutes, separated by SDS-PAGE, and transferred. Primary antibodies used at 1∶2000 dilution include rat anti-HA (Roche), RR-14, and TAp63. Secondary antibodies used at a 1∶5000 dilution were immunoPure goat anti-rat and goat anti-rabbit horseradish peroxidase-conjugated IgG (Pierce, Rockford, IL). Chemiluminescence detection of horseradish peroxidase-conjugated secondary antibodies was performed using the horseradish peroxidase stabilizer kit from KPL (Gaithesburg, ND).

### Cell culture

HaCaT cells were maintained in DMEM supplemented with 10% fetal bovine serum and 100 U/ml penicillin and 100 µg streptomycin. Cells were routinely passaged at 90% confluency. HeLa (human cervical adenocarcinoma) cells were grown in DMEM supplemented with 10% fetal bovine serum, 100 U/ml penicillin, and 100 µg/ml streptomycin. Ptk2 (rat kangaroo kidney epithelial) cells were grown in Modified Eagle's Medium with 10% fetal bovine serum, 1% Modified Eagle's Medium Non-Essential Amino Acid solution, 100 U/ml penicillin, and 100 µg/ml streptomycin. A spontaneously immortalized mouse keratinocyte (MK) cell line was grown as described previously [45].

### ChIP experiments

ChIP experiments with anti-p63 antibodies using cells in culture have been described previously [23], [25]. Primers used to amplify the Hs II region of the human *K5* gene were forward 5′-CTGGCAGATGTGCATGCCAGCTGC-3′ and reverse 5′-CTCTTTGGCCTGGGCAGGACTCTG-3′. Human glyceraldehydes-3-phosphate dehydrogenase control primers were forward 5′- GAGTACGCTGCAGGGCCTCACTCCTTTTGC-3′ and reverse 5′- CATGCCAGTGAGCTTCCCGTTCAGCTCAG-3′. The following primers were used to amplify the Hs II region of the mouse *K5* gene, forward 5′-GCAGAAGAGTCGTCTGGAAGC-3′ and reverse 5′-GCTGTGTTCCAGCAAGTT-3′. Mouse glyceraldehydes-3-phosphate dehydrogenase control primers were forward 5′- GCTAGGACTGGATAAGCAGG-3′ and reverse 5′- GGTCCGGCTTGCACACTTC-3′.

## Supporting Information

Figure S1 Genomic sequence conservation of K5. Upper panel shows a schematic of the various DNAse I Hs previously identified in the human K5 gene [6]. The sequence of Hs II from various species was obtained from the respective genome database and aligned. Hs II reveals high sequence conservation among seven species as indicated. The p63-response element is denoted by a horizontal bar.
(0.13 MB TIF)

Figure S2 Hs II of the K5 gene acts as an enhancer and is activated by various p63 isoforms in reporter gene assays in mouse keratinocytes. In the upper panel, a luciferase construct containing Hs II upstream of the heterologous TK promoter (Hs IITK), shows a three fold activation over the TK promoter alone (TK). In contrast, a luciferase construct containing the Hs II upstream of the human K5 promoter (Hs II K5), showed a 7.5 fold higher activation as compared to the K5 promoter alone (K5). In the lower panel, the Hs II K5 construct was co-transfected with expression plasmids encoding various isoforms of p63 into Ptk2 cells. Luciferase values were determined and normalized against β-galactosidase values. The corrected luciferase values for cells transfected with empty vector pCMV-HA were set at 1.
(0.04 MB TIF)

Figure S3 ΔNp63 can induce de novo expression of the keratinocyte differentiation markers K1 and K10 in single-layered lung epithelia. Lung tissue sections from E18.5 ΔNp63α BG animal reveals de novo expression of K1 and K10 (green) as compared to control animals. Transgene (HA) expression is shown in red. Scale bar: 25 µm.
(0.13 MB TIF)

Figure S4 Ectopic Expression of ΔNp63α can partially rescue the p63 null phenotype. Top left panel shows H&E staining of p63−/− and p63−/−,ΔNp63α rescued animals. Inset is a higher magnification demonstrating the partial rescue of the epidermis in the transgenic animals. Remaining panels illustrate immunofluorescence staining using various antibodies as indicated (in green) in 20× magnification. The antibodies used were against ΔNp63 (RR-14) and p63α (H-129). White arrowhead shows filaggrin expression in the epidermis of the ΔNp63α/p63−/− animals. White hashed line demarcates the dermal epidermal boundary. Scale bar: 50 µm.
(0.50 MB TIF)
