# Supplementary figures and images for: An Active Role of the ΔN Isoform of p63 in Regulating Basal Keratin Genes K5 and K14 and Directing Epidermal Cell Fate
Source: PLoS One. 2009 May 20;4(5):e5623. doi: 10.1371/journal.pone.0005623 (PMC2680039; doi:10.1371/journal.pone.0005623)

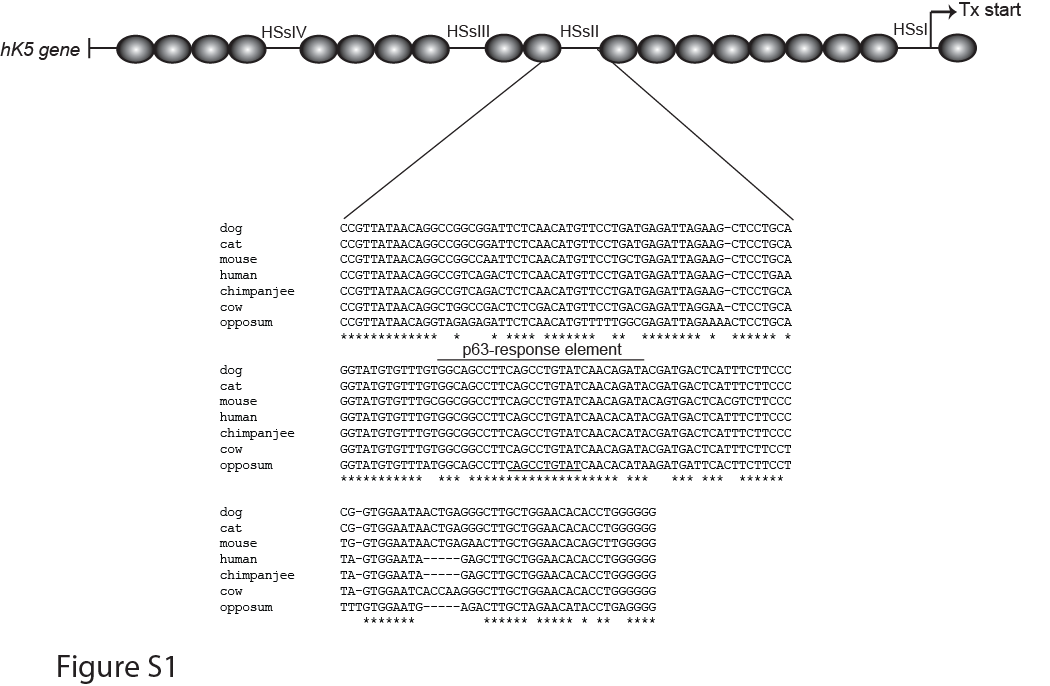

Supplement: Figure S1 — Genomic sequence conservation of K5. Upper panel shows a schematic of the various DNAse I Hs previously identified in the human K5 gene [6]. The sequence of Hs II from various species was obtained from the respective genome database and aligned. Hs II reveals high sequence conservation among seven species as indicated. The p63-response element is denoted by a horizontal bar. (0.13 MB TIF) [file pone.0005623.s001.tif]

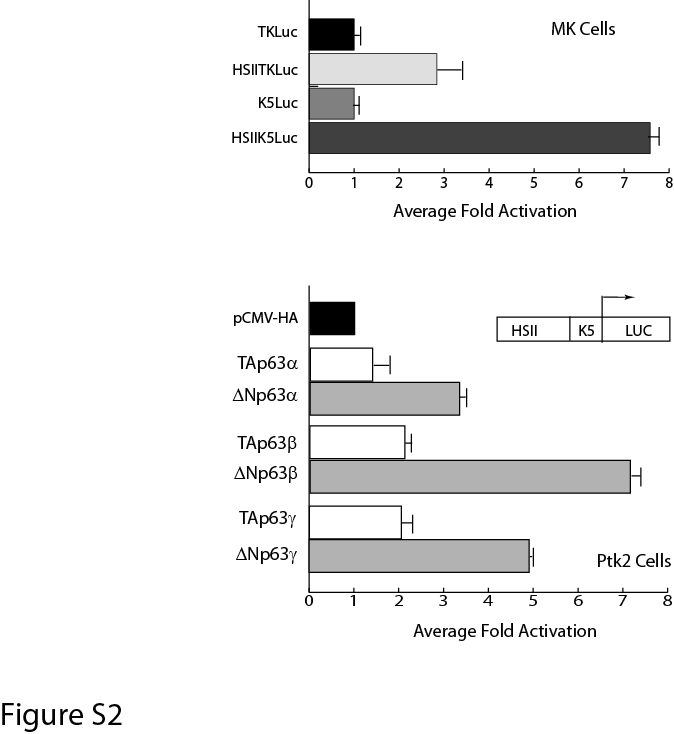

Supplement: Figure S2 — Hs II of the K5 gene acts as an enhancer and is activated by various p63 isoforms in reporter gene assays in mouse keratinocytes. In the upper panel, a luciferase construct containing Hs II upstream of the heterologous TK promoter (Hs IITK), shows a three fold activation over the TK promoter alone (TK). In contrast, a luciferase construct containing the Hs II upstream of the human K5 promoter (Hs II K5), showed a 7.5 fold higher activation as compared to the K5 promoter alone (K5). In the lower panel, the Hs II K5 construct was co-transfected with expression plasmids encoding various isoforms of p63 into Ptk2 cells. Luciferase values were determined and normalized against β-galactosidase values. The corrected luciferase values for cells transfected with empty vector pCMV-HA were set at 1. (0.04 MB TIF) [file pone.0005623.s002.tif]

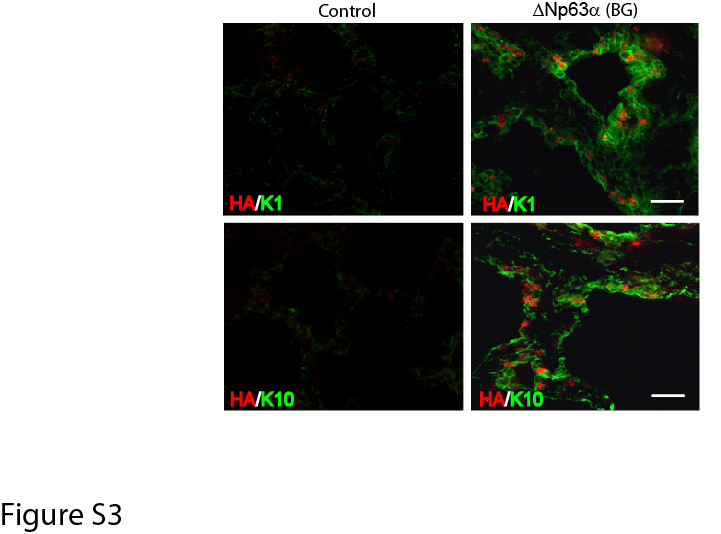

Supplement: Figure S3 — ΔNp63 can induce de novo expression of the keratinocyte differentiation markers K1 and K10 in single-layered lung epithelia. Lung tissue sections from E18.5 ΔNp63α BG animal reveals de novo expression of K1 and K10 (green) as compared to control animals. Transgene (HA) expression is shown in red. Scale bar: 25 µm. (0.13 MB TIF) [file pone.0005623.s003.tif]

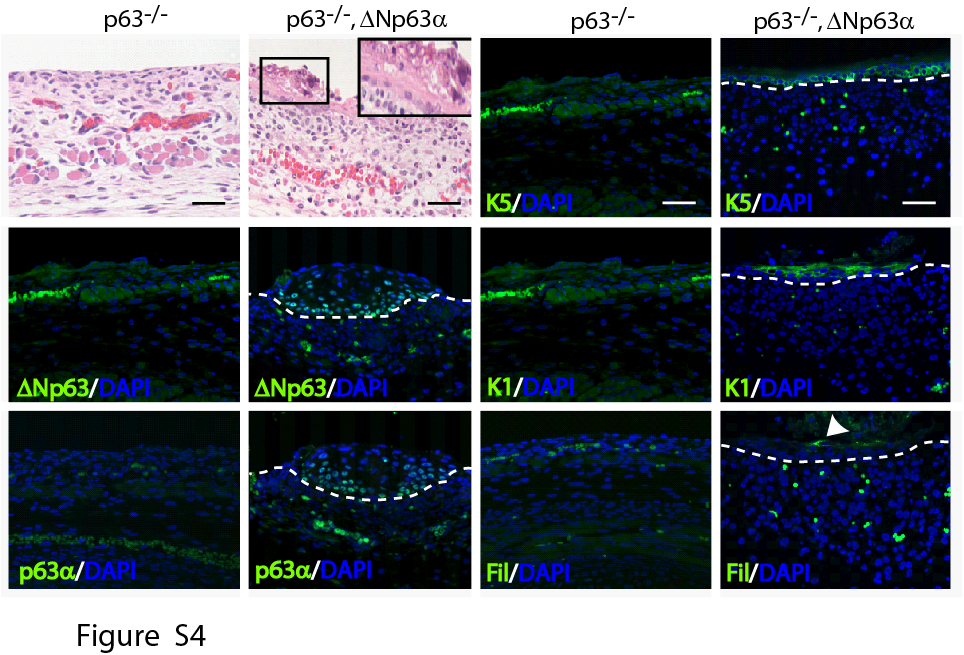

Supplement: Figure S4 — Ectopic Expression of ΔNp63α can partially rescue the p63 null phenotype. Top left panel shows H&E staining of p63−/− and p63−/−,ΔNp63α rescued animals. Inset is a higher magnification demonstrating the partial rescue of the epidermis in the transgenic animals. Remaining panels illustrate immunofluorescence staining using various antibodies as indicated (in green) in 20× magnification. The antibodies used were against ΔNp63 (RR-14) and p63α (H-129). White arrowhead shows filaggrin expression in the epidermis of the ΔNp63α/p63−/− animals. White hashed line demarcates the dermal epidermal boundary. Scale bar: 50 µm. (0.50 MB TIF) [file pone.0005623.s004.tif]
